# Loss of EGFR-ASAP1 signaling in metastatic and unresectable hepatoblastoma

**DOI:** 10.1038/srep38347

**Published:** 2016-12-02

**Authors:** Sarangarajan Ranganathan, Mylarappa Ningappa, Chethan Ashokkumar, Brandon W. Higgs, Jun Min, Qing Sun, Lori Schmitt, Shankar Subramaniam, Hakon Hakonarson, Rakesh Sindhi

**Affiliations:** 1 Children’s Hospital of Pittsburgh of University of Pittsburgh Medical Center, 4401 Penn Avenue, Department of Pathology, Pittsburgh, PA 15224, USA; 2 Children’s Hospital of Pittsburgh of University of Pittsburgh Medical Center, 4401 Penn Avenue, Pediatric Abdominal Transplant Surgery, Pittsburgh, PA 15224, USA; 3 Department of Bioengineering, University of California San Diego, LA Jolla, CA, USA.; 4 Center for Applied Genomics, Children’s Hospital of Philadelphia, 1216 E. Abramson’s Research Center, 34th and Civic Center Blvd., ARC 1216E, Philadelphia, PA. 19104, USA

## Abstract

Hepatoblastoma (HBL), the most common childhood liver cancer is cured with surgical resection after chemotherapy or with liver transplantation if local invasion and multifocality preclude resection. However, variable survival rates of 60–80% and debilitating chemotherapy sequelae argue for more informed treatment selection, which is not possible by grading the Wnt-β-catenin over activity present in most HBL tumors. A hypothesis-generating whole transcriptome analysis shows that HBL tumors removed at transplantation are enriched most for cancer signaling pathways which depend predominantly on epidermal growth factor (EGF) signaling, and to a lesser extent, on aberrant Wnt-β-catenin signaling. We therefore evaluated whether EGFR, ASAP1, ERBB2 and ERBB4, which signal downstream after ligation of EGF, and which show aberrant expression in several other invasive cancers, would also predict HBL tumor invasiveness. Immunohistochemistry of HBL tumors (n = 60), which are histologically heterogeneous, shows that compared with well-differentiated fetal cells, less differentiated embryonal and undifferentiated small cells (SCU) progressively lose EGFR and ASAP1 expression. This trend is exaggerated in unresectable, locally invasive or metastatic tumors, in which embryonal tumor cells are EGFR-negative, while SCU cells are EGFR-negative and ASAP1-negative. Loss of EGFR-ASAP1 signaling characterizes undifferentiated and invasive HBL. EGFR-expressing HBL tumors present novel therapeutic targeting opportunities.

Although rare, hepatoblastoma (HBL) is the most common liver cancer during childhood, and accounts for nearly a tenth of all pediatric liver transplants in the United States^1^,^2^. This public health impact can be alleviated if chemotherapy and surgical options are targeted to tumor biology. This task is challenging because HBL presents heterogeneity on several fronts. Tumor histology shows varying mixtures of the more differentiated fetal, less differentiated embryonal cells and undifferentiated small cells (SCU)^3^. Chemotherapy followed by local resection can be curative^4^,^5^. However, not all tumors are chemosensitive and liver transplantation is needed if the tumor cannot be resected surgically because of size, multifocality or proximity to or invasion of large central vessels^6^. Survival rates vary from 60–80% and chemotherapy sequelae such as deafness are common among survivors, suggesting room for improvement^5^,^6^,^7^. The pathognomonic overexpression of β-catenin in HBL is not prognostic. Further, survival is not reliably predicted by β-catenin mutations, which are present in the majority but not all tumors, or the intracellular location of the corresponding protein^8^,^9^,^10^,^11^. Because induced overexpression of β-catenin is not oncogenic by itself in the animal models, a ‘second hit’ is strongly implicated^12^. Potential second hits include several tumor-associated markers and mechanisms such as the Yes-associated protein 1 (Yap1), c-Myc, hepatocyte growth factor (HGF)/c-Met, NOTCH, MAP kinase, cyclin D, ki-67, telomerase reverse transcriptase (TERT), and RASS1FIA methylation status^13^,^14^,^15^,^16^,^17^,^18^,^19^,^20^. This list may be incomplete because copy number gains are seen in several chromosomes in the tumor exome of surgically resected HBL^21^,^22^,^23^. HBL requiring transplantation may also contain additional unique chromosomal changes (personal communication). The association of HBL with low birth weight and prematurity suggests complex disease causation^24^.

Novel candidates for intervention have been identified for complex disease traits and other embryonal tumors like neuroblastoma using unbiased genome-wide searches^25^,^26^. Because of its developmental origin and its syndromic associations, HBL is also amenable to such approaches. HBL is associated with other birth defects, evident on prenatal ultrasound in some cases, and mostly seen in children less than five years old^27^,^28^. A familial basis is also suggested by a 1% incidence in children with a family history of familial adenomatosis polyposis, where it is associated with mutations in the *APC* gene^29^. Population level susceptibility or environmental factors are also likely because of a 2–4-fold higher tumor incidence in Taiwan and China, and a higher incidence in children with Beckman-Weidemann syndrome, where widespread tissue overgrowth is associated with benign and malignant tumors^30^,^31^. The incidence of HBL is 3–4 per million in North America^2^.

In this study, we use whole transcriptome analysis of invasive and locally unresectable HBL tumors which required transplantation to generate a hypothesis for HBL pathogenesis. For hypothesis testing, we evaluate whether tumor de-differentiation and outcomes are associated with differential immunostaining of the candidate genes and related pathway members in archived residual formalin-fixed-paraffin-embedded (FFPE) HBL tumor tissue. Because resectable tumors outnumber those requiring transplantation four-fold, immunostaining studies were performed in samples from an expanded cohort of affected children treated with and without liver transplantation^6^.

## Results

### Human Subjects

Children with HBL included 23 who received liver transplantation for unresectable tumors, 40 in whom the tumor was removed with surgical resection, and one who only received diagnostic biopsy. Available information for 63 of 64 children revealed mean (SEM) age of 30.4 (3.6) months (median 24 months, range: 6–156), and male: female distribution of 35:28. No follow up was available in four of 64 children, three of whom had primary resection, and the fourth only received a diagnostic biopsy. Sixty children are alive and disease-free at last follow-up. Five children died, one due to sepsis, and four due to metastatic or recurrent disease, two after surgical resection and two after liver transplantation. A segmental lung metastases was successfully resected 27 months after transplantation in another child who is disease-free 7 years after transplantation.

**Figure 1:**
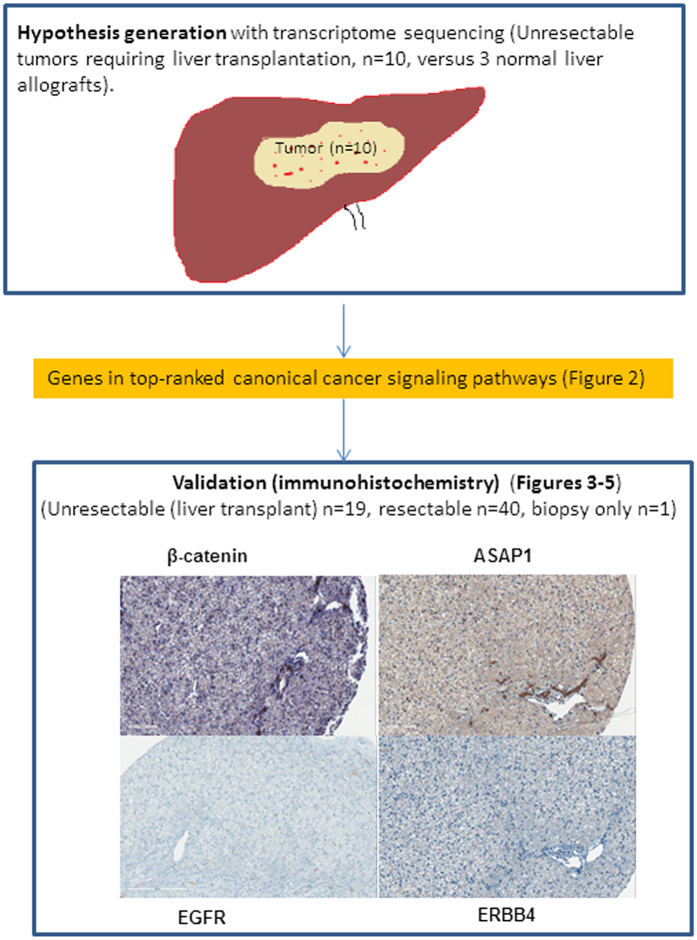
Study design. Hypothesis-generation was conducted with whole transcriptome analysis of liver tumor samples from children with unresectable HBL requiring liver transplantation. Top-ranked canonical cancer signaling pathways were identified from differentially expressed genes in HBL tumor compared with normal liver tissue. Validation of selected members (*EGFR, ASAP1, ERBB4,* and β-catenin) in candidate pathways (EGF/ERBB) and their relationship to β-catenin was conducted with immunohistochemistry of residual FFPE tissue from children with resectable and unresectable tumors. β -catenin stain shows membranous staining of normal hepatocytes and biliary epithelial cells in normal liver control (β- catenin ×200). ASAP1 stain shows strong staining in biliary epithelial cells and no staining in normal hepatocytes, which give a faint background blush (x20 digital whole image scan equivalent of ×200). EGFR shows no staining in hepatocytes with normal biliary cells showing faint membranous staining (x20 digital whole image scan equivalent of ×200). ERBB4 stain is negative in hepatocytes and biliary cells in normal liver (x20 digital whole image scan equivalent of ×200). FFPE = formalin-fixed paraffin-embedded.

The distribution of subjects from whom available liver tissue was used for gene expression analysis with mRNA sequencing, and residual FFPE tumor tissue was available for immunohistochemistry is shown in the study flow diagram in Fig. 1. Studies were conducted in those children who were recruited in prospective University of Pittsburgh protocol IRB#0405628. Immunohistochemistry was performed under protocol IRB# 09030166.

For immunohistochemistry studies on available residual FFPE tissue, 60 children were grouped as having resectable (n = 39) or unresectable (n = 21) tumors. Unresectable tumors were those requiring LTx (n = 19) and/or those with metastases (n = 3), 2 after surgical resection and 1 with liver transplantation. The child with successful resection of post-transplant metastasis is included in the transplant cohort.

### Aberrant EGFR and Wnt-β-catenin signaling in the whole tumor transcriptome.

**Figure 2:**
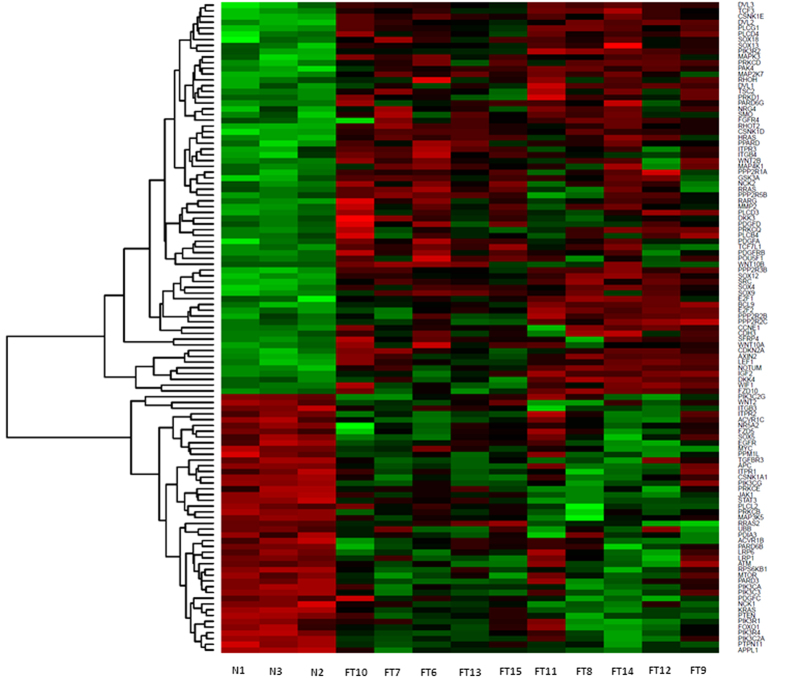
Heatmap shows hierarchical clustering of selected differentially expressed genes in HBL tumor tissue from 10 children who received liver transplantation (FT7–FT16) compared with liver tissue from normal liver allograft donors (N1, N2, N3). The selected genes participate in the top-ranked glioblastoma multiforme signaling and Her-2 breast cancer signaling pathways, which are enriched in the HBL tumor transcriptome. Because these pathways are largely made up of EGF/ERBB signaling genes, and to a lesser extent, Wnt-β-catenin signaling genes, differentially expressed genes belonging in these constituent pathways are also included. Color legend: red-overexpression, green underexpression.

 We first characterized the whole tumor transcriptome with mRNA sequencing to generate a hypothesis. We characterized the tumor transcriptome of 10 children with HBL who received transplantation and the transcriptome of liver tissue from three healthy donor allografts. Genes with an adjusted p < 0.05 and |fold change| > 2 were retained for further analysis (Supplementary Table S1). We found that of 6563 differentially expressed genes with q-values < 0.05 in HBL tumors, 4327 are associated with cancer, which emerged as the top-ranked disease (p-value range 6.58E-04–6.57E-109, IPA, Redwood, CA). Further, these differentially expressed genes were enriched for 499 canonical pathways (Supplementary Table S2) including well-known cancer signaling pathways. The two highest ranked cancer-related pathways were glioblastoma multiforme signaling (GBM, p = 1.5E-04) and Her2 breast cancer signaling (p = 9.48E-04). We compared gene lists ascribed to these pathways in the tumor transcriptome with those for EGF or ERBB signaling and Wnt β-catenin signaling, which were also enriched in the tumor transcriptome (Supplementary Table S3). The ERBB group of receptors includes four receptors ERBB1–4 of which ERBB1 is also called EGFR^32^,^33^. We found that EGF/ERBB signaling genes made up a larger proportion of cancer signaling genes in GBM and Her2 breast cancer signaling pathways, compared with those in the Wnt-β-catenin signaling pathway (Supplementary Table S3). qRT-PCR confirmed changes in gene expression for selected genes in the EGF/ERBB *(EGFR, PI3C2G, MYC, PLCG1)* and Wnt *(Wnt2A, AXIN2, SRC)* signaling pathways (Supplementary Table S4). This suggested that EGF-dependent cancer pathways were enriched the most in the transcriptome of HBL tumors requiring transplantation. Hierarchical clustering analysis of gene expression changes in these pathways demonstrates these differences in the tumor transcriptome of each subject compared with normal liver tissue (Fig. 2).

### Impaired EGFR-ASAP1 signaling in less differentiated, unresectable HBL.

 Therefore, we evaluated the relationship between the known Wnt-β-catenin and EGFR signaling pathways in HBL with immunohistochemistry. EGFR and its effector, ASAP1 are overexpressed and associated with increased invasiveness in several cancers, and also have dominant roles in GBM and breast cancer signaling^33^,^34^,^35^,^36^,^37^,^38^,^39^. Both GBM and breast cancer can also overexpress β-catenin^40^,^41^. Combined activation of the Wnt-β-catenin and EGFR pathways is also implicated in hepatocellular carcinoma^42^,^43^. EGFR, also known as ERBB1, can have an inverse relationship to other members of the EGFR family, ERBB2–4, ERBB-3 and ERBB-4 in breast cancer^44^. ERBB4 antagonizes the nuclear effects of EGFR signaling^45^.

We performed immunostaining of EGFR, ASAP1, ERBB2, ERBB4 and β-catenin in HBL tumor tissue from an expanded cohort of children treated with liver transplantation (n = 19) or with surgical resection (n = 40) and biopsy (n = 1). The 19 children with transplantation included six whose tumors were subjected to transcriptome sequencing (Fig. 1). Staining intensity was graded as 0–3 with 3 representing intense staining for each marker (Grade 0 = no staining; 1 = up to 10% staining; 2 = 10–50% staining of cells; and 3 => 50% staining of tumor cells).

**Figure 3:**
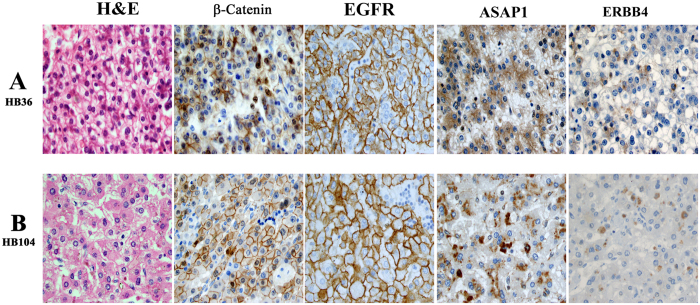
Fetal HBL tumor cells with abundant eosinophilic cytoplasm (hematoxylin-eosin, H&E) from resectable (panel A) and unresectable HBL which required liver transplantation (panel B). Fetal HBL tumor cells show nuclear, cytoplasmic and membranous β-catenin staining, membranous EGFR staining, and intense ASAP1 staining. Also shown is patchy ERBB4 staining of fetal cells from another HBL tumor (panel B). (All images ×400).

**Figure 4:**
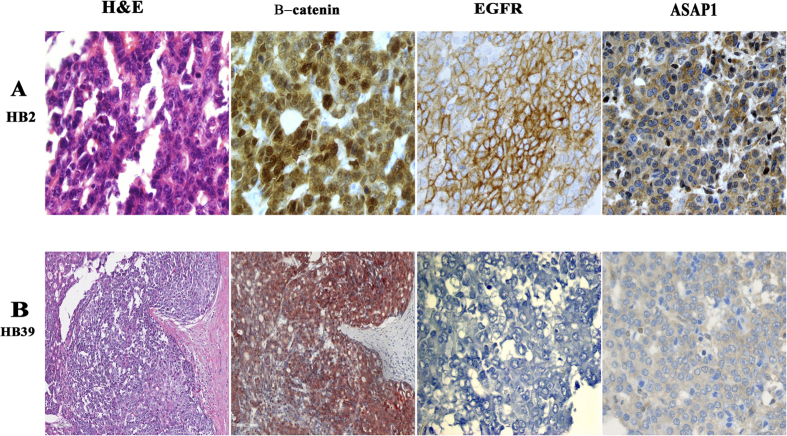
Embryonal HBL tumor cells with large nuclei and scant cytoplasm from a resectable (panel A, x400) and unresectable (panel B, x200) tumor which required liver transplantation. Also shown are corresponding immunostains for β-catenin, EGFR and ASAP1. (panel B – β-catenin x200; all other images × 400).

**Figure 5:**
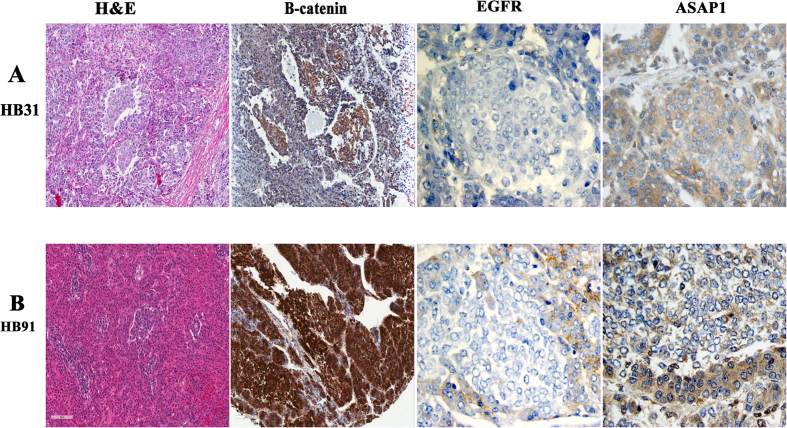
SCU HBL tumor cells with minimal cytoplasm and lightly stained nuclei from a resectable (panel A, x200) and unresectable (panel B, x200) HBL tumor which required liver transplantation. Also shown are corresponding immunostains for β-catenin (x200), EGFR and ASAP1 (x400).

Nuclear β**-**catenin staining was present in all embryonal and all SCU tumor cells, and in fetal tumor cells from all except a fifth of all HBL tumors (Table 1). Fetal tumor cells also showed variable/less intense staining, with more membranous and cytoplasmic staining (Fig. 3). The EGFR family member, ERBB4, was expressed in fetal tumor cells from 60% of tumors, embryonal cells from 17% of HBL tumors, but absent from all SCU cells (p < 0.001, chi-square test). The less differentiated embryonal and SCU tumor cells also demonstrated a similar loss of immunostaining for the other EGFR pathway members, EGFR and ASAP1 (Figs 4 and 5). EGFR immunostaining was present in fetal tumor cells from three-fourths of HBL tumors, and embryonal cells from half of all HBL tumors, but absent from SCU cells from all HBL tumors (p < 0.001). ASAP1 immunostaining was present in fetal and embryonal cells from nearly all HBL tumors but in SCU cells from only 40% of all HBL tumors (p < 0.001). ERBB2 was not expressed in any cell type in any of the tumors.

Because the expression of EGFR pathway members was progressively lost with less differentiated tumor cells, we asked whether this trend would be more pronounced among invasive tumors, i.e., those unresectable tumors that required liver transplantation and/or were metastatic, within each tumor cell type. Consistent with previous reports, β-catenin staining intensity did not differ significantly between resectable and unresectable tumors within a tumor cell type, fetal, embryonal or SCU (Table 2). Although ERBB4 expression decreased in fetal and embryonal cells from unresectable tumors compared to resectable tumors, these differences failed to achieve significance. Only a third of unresectable tumors contained embryonal cells expressing EGFR, compared with 70% of resectable tumors (p = 0.047). No significant differences were seen in ASAP1 expression in embryonic cells from resectable and unresectable tumors. SCU cells, which were all EGFR negative, also failed to express ASAP1 in unresectable tumors. In comparison, ASAP1 immunostaining was present in SCU cells from two-thirds of resectable tumors (p = 0.035).

## Discussion

Using whole tumor transcriptome sequencing, we show for the first time that the transcriptome of HBL tumors which required liver transplantation is enriched most for cancer signaling pathways, in which the larger proportion of differentially expressed genes participate in EGF and ERBB signaling (Supplementary Tables S2 and S3). Both top-ranked cancer signaling pathways, glioblastoma multiforme signaling and Her-2 breast cancer signaling, also contain some but not all differentially expressed Wnt-β-catenin signaling genes present in the HBL tumor transcriptome. Abnormal EGF signaling has a major role in GBM and breast cancer, where Wnt-β-catenin signaling is also increasingly implicated^40^,^41^. Therefore, our findings suggest that in addition to the accepted role of enhanced Wnt-β-catenin signaling in HBL, aberrant EGF-dependent cancer signaling likely contributes to HBL tumorigenesis. Abnormal EGFR signaling together with dysregulated Wnt, JAK/STAT and mTOR signaling pathway genes has been observed in transcriptome sequencing of the HepG2 hepatoblastoma cells^46^. In our study, the corresponding canonical cancer pathways in the HBL tumor transcriptome are GBM, Her-2 breast cancer, EGF and ERBB signaling (EGFR), Wnt-β-catenin signaling (Wnt), STAT3 and JAK/STAT signaling (JAK/STAT), and mTOR signaling pathways (mTOR, Supplementary Table S2).

Interestingly, HBL tumors show downregulation of the EGFR gene in contrast with upregulation seen in several invasive cancers including hepatocellular carcinoma. This may be a unique feature of HBL tumors not reported previously, but supported by the decreased expression of EGFR by western blot in the HepG2 hepatoblastoma cell line, compared with four other hepatocellular cancer cell lines, HLF, Huh7, Hi7, and PLC/PRF/5^47^. In another study, the transcriptome of the HepG2 cell line and normal hNHEP hepatocytes was compared using cDNA microarrays^48^. Several up- and downregulated genes were identified along with differential scores. On pathway analysis of these differentially expressed genes, we have found enrichment of the same canonical cancer signaling pathways as those in our study (Supplementary Table S5). Gene expression analysis with qRT-PCR has also shown *EGFR* gene upregulation in the more differentiated types of HBL tumor cells, and the lowest expression in HBL tumors containing predominantly small undifferentiated tumor cells^49^. It is not clear whether these tumors were obtained from children who received surgical resection or transplantation. Also, the relationship of the various members of the EGFR family to Wnt-β-catenin signaling is not known within the individual HBL tumor cell types, fetal, embryonal and SCU. Whether these relationships determine tumor behavior, localized and resectable versus locally invasive and therefore requiring transplantation is of great interest. Protein-protein interactions between EGFR and β-catenin can promote tumorigenesis via cross-talk between these two pathways^50^.

Breast cancer invasiveness has been linked to ASAP1 activation by ARF6, which is in turn activated by EGFR ligation to GEP100^35^. EGFR is negatively regulated by another EGFR family member, ERBB4, in breast cancer^45^. This activation sequence may be unique to invasive cancer because normal liver and breast tissue shows limited expression of EGFR and ERBB4 and widespread expression of ASAP1 (www.proteinatlas.org).

Immunohistochemistry studies extended to HBL tumor tissue from subjects treated with surgical resection or transplantation confirm that aberrant EGFR-ASAP1 signaling is associated with loss of differentiation and increased invasiveness in HBL. This pattern may also predict outcomes. The less differentiated embryonal and SCU cells lose the ability to express EGFR pathway members compared with the well-differentiated fetal cells. ERBB4, which is expressed in fetal cells from 60% of HBL tumors, is expressed in embryonal cells from only 17% of HBL tumors, and is not expressed in SCU cells from all HBL tumors. EGFR, which is expressed in fetal cells from three-fourths of HBL tumors, is expressed in embryonal cells from half of all HBL tumors, and is not expressed in SCU cells from any HBL tumor. ASAP1, which is expressed in fetal and embryonal cells from 96% of all tumors is expressed in SCU cells from 40% of all HBL tumors. This hierarchical loss of expression of EGFR pathway members is magnified in metastatic tumors and in invasive (unresectable) HBL tumors requiring liver transplantation. Only a third of embryonal cells from unresectable HBL expressed EGFR compared with 70% from resectable tumors (p = 0.047). No such differences were evident for ERBB4 or ASAP1 expression. SCU cells which are EGFR-negative also failed to express ASAP1 when obtained from unresectable HBL. In contrast, ASAP1 was expressed in SCU cells from two-thirds of resectable tumors (p = 0.035). Therefore, loss of EGFR expression in embryonal cells and loss of EGFR and ASAP1 expression in SCU cells may characterize the HBL tumor likely to need liver transplantation, or metastasize. These findings potentially argue for an aggressive search for advanced local or systemic disease. In selected HBL cases with a predominantly fetal cell component, intense EGFR expression may present a rationale for using EGFR inhibitors as an alternative to cisplatin and Adriamycin, which can be ototoxic and cardiotoxic, respectively, and are current first-line drugs for HBL^4^,^44^,^45^. Unresectable and resectable tumors did not show significant differences in ERBB4-expressing fetal cells.

Our findings strongly suggest that EGFR-ASAP1 signaling contributes to HBL tumorigenesis in a subset of patients^33^,^34^,^35^,^36^,^37^,^38^,^39^. Because several genes and pathways have been shown by others to synergize with the Wnt-β-catenin pathway in HBL, it is likely that EGFR-ASAP1 signaling is another such pathway, which modulates Wnt-β-catenin signaling in HBL in a subset of affected children. A distinct feature warranting explanation is loss of ASAP1 and EGFR expression in unresectable/invasive HBL, rather than the increased expression seen in other invasive cancers. This feature may be unique to embryonal cancers such as HBL because EGFR signaling antagonizes the effects of Wnt-β-catenin in developing fetal cells^51^,^52^. Whether loss of EGFR signaling augments Wnt-β-catenin-mediated invasiveness and loss of differentiation in HBL will require further study.

A limitation of our study is that gene expression analysis and immunohistochemistry have been performed in modest sub-cohorts of the rare test population, with variable overlap in subject composition. However, hypothesis-generation occurred under identical conditions in HBL tumors explanted at the time of transplantation, with all children having received recommended chemotherapy^3^,^4^. Further, the relationship of EGFR family members to tumor behavior was evaluated in two complementary settings: in HBL tumor cell types organized by increasing de-differentiation, and in tumors classified by whether they were locally resectable with surgery, or required removal by transplantation because of extent, multifocality or proximity to blood vessels. That our findings agree in part with work done in the HepG2 hepatoblastoma cell line, and in selective gene expression studies in explanted HBL tumors, both of which show reduced EGFR protein expression globally, or reduced EGFR gene expression in the least differentiated cells is also reassuring. In this regard, enrichment of genes for GBM and Her2 breast cancer signaling in the whole tumor transcriptome in our studies and those from the HepG2 cell line are striking, because aberrant EGFR signaling, and to a lesser extent, Wnt-β-catenin signaling is implicated in both of these tumors. In contrast, Wnt-β-catenin, but not EGFR signaling has been evaluated extensively in HBL tumorigenesis. Protein expression characterized with immunohistochemistry confirms that both pathways are also involved in HBL. This immunohistochemistry panel resolves marker expression by tumor cell type and has the potential for assigning risk to each HBL tumor. If validated, the prognostic implications can be significant. Less toxic chemotherapy may be selected for those localized tumors amenable to local resection and in which EGFR signaling is intact. In contrast, tumors in which constituent cell types show loss of EGFR signaling may argue for a more aggressive search for metastases or removal by transplantation, if, for example, multiple localized HBL lesions are seen on imaging studies.

In summary, we report the novel finding from children with unresectable HBL requiring liver transplantation that aberrant EGFR-ASAP1 signaling enhances HBL invasiveness and is likely one of many second hits for HBL tumorigenesis. Tumor immunohistochemistry of an expanded cohort of resectable and unresectable HBL shows progressive loss of EGFR-ASAP1 signaling in less differentiated HBL tumor cells. This loss is more pronounced in and may predict unresectable/metastatic HBL. If validated prospectively, the novel immunohistochemistry panel where the role of EGFR-ASAP1 signaling was confirmed in individual tumor cells offers the potential for individualized risk-stratification of each HBL tumor.

## Methods

### Human Subjects

All studies were performed after informed consent and carried out in accordance with the approved guidelines of the Institutional Review Board of the University of Pittsburgh’s (IRB#0405628 for children, and IRB#0507027 for parents). All experimental protocols were approved by the Institutional Review Board of the University of Pittsburgh.

### Samples

Using previously described methods, RNA was extracted from pre-perfusion liver allograft biopsies obtained at LTx from children with HBL^53^.

### Transcriptome sequencing

Total RNA was isolated from fresh frozen liver tissues with hepatoblastoma (HBL) using the RNA easy RNA isolation kit (Qiagen, Valencia, CA). RNA sequencing libraries were prepared from fresh frozen liver with HBL RNA (1 μg) using a SureSelect Strand Specific RNA Library Preparation kit (Product#G9691A, Agilent Technologies, Santa Clara, CA) accordingly to the manufacture’s protocol. Libraries were sequenced with HiSeq2500 system (Illumina) using TruSeq SBS Kit v3 - HS (200-cycles) and TruSeq PE Cluster Kit v3 (cat# FC-401–3001 and Cat# PE-401–3001, Illumina, San Diego, CA).

For mRNA sequencing data, 100mer paired-end reads were mapped to the human genome (UCSC hg19; Feb 2009 release; Genome Reference Consortium GRCh37) using HISAT2 (v2.0.1)^54^. Transcript counts were calculated and normalized with the human reference gtf annotation file (GRCh37.68) using DESeq2 (v1.6.3)^55^. A Welch’s modified t-test was used to calculate p-values between normal and hepatoblastoma patient specimens and subsequently adjusted with the qvalue package^56^. Genes with an adjusted p < 0.05 and |fold change|>2 were retained (6,563 genes). The heatmap was generated in R.

### Gene expression analysis

Total RNA (1 μg) isolated from fresh frozen liver tissues with hepatoblastoma (HBL) and normal adult donor liver tissue samples was reverse-transcribed to cDNA using high capacity cDNA reverse transcription kit (Thermo Fisher Scientific, Waltham, MA). Quantitative real time PCR was performed in Taqman gene expression master mix (Thermo Fisher Scientific, Waltham, MA) using Taqman gene expression assays (Thermo Fisher Scientific) for *EGFR* (Assay ID: Hs01076090_m1), *AXIN2* (Assay ID: Hs00610344_m1), *MYC* (Assay ID: Hs00153408_m1), *PIK3C2G* (Assay ID; Hs00362135_m1), *WNT2* (Hs00608224_m1). *PLCG1* (Assay ID: Hs01008225_m1), *SRC* (Assay ID: Hs01082246_m1). Quantitative PCR was performed on a model 7300 real-time PCR system (Thermo Fisher Scientific, Waltham, MA), with 10 minutes incubation at 95 °C to activate AmpliTaq Gold DNA polymerase; this was followed by 40 cycles with 15 seconds at 95 °C and 1 minute at 60 °C for each cycle. Because of variable expression of housekeeping genes in tumor tissue^57^, we used direct comparison of threshold Cycle value (Ct) in HBL liver tissue and normal liver tissue.

### Tissue immunohistochemistry

Formalin fixed, paraffin embedded tissues from 63 children, 40 with surgical resection, 22 with LTx and one with biopsy only were prepared into a tissue microarray and stained for hematoxylin-eosin and for immunohistochemical stains on the Ventana BenchMark Ultra automated staining platform using the following protocols:

### EGFR immunostaining

Slides were digested with Protease I (proprietary, Ventana Medical Systems Inc., Tucson, AZ) for 8 minutes and stained using a mouse monoclonal primary antibody (Ventana Medical Systems Inc., Tucson, AZ, 790–2988). OptiView DAB detection kit (proprietary, indirect, biotin free system, Ventana Medical Systems Inc., Tucson, AZ) was used for primary antibody detection.

### ErbB4 immunostaining

Slides were pretreated with ultraCC1 (proprietary, Ventana Medical Systems Inc., Tucson, AZ) for 64 minutes and stained using a rabbit monoclonal primary antibody (Abcam, Cambridge, MA, ab68478, 1:10). UltraView DAB detection kit (proprietary, indirect, biotin free system, Ventana Medical Systems Inc., Tucson, AZ) was used for primary antibody detection.

### ASAP1 immunostaining

Slides were pretreated with ultraCC1 (proprietary, Ventana Medical Systems Inc., Tucson, AZ) for 36 minutes and stained using a rabbit polyclonal primary antibody (Abcam, Cambridge, MA, ab11011, 1:1000). UltraView DAB detection kit (proprietary, indirect, biotin free system, Ventana Medical Systems Inc., Tucson, AZ) was used for primary antibody detection.

β-catenin (VentanaMed Systems Inc, Tucson, AZ) was used as a prediluted antibody after antigen retrieval.

All slides were counterstained with hematoxylin and routinely dehydrated, cleared, and cover- slipped in resinous mounting media. Staining intensity for each of the abovementioned markers was evaluated in each of four different HBL tumor components, fetal (n = 51), embryonal (n = 29), and SCU (n = 10). The less common mesenchymal cell type (n = 4) was not analyzed. The distribution of staining was also noted (membranous versus cytoplasmic versus nuclear) for all stains. Stain controls, positive and negative, were used for all stains and runs. Between-group comparisons were performed with chi-square test.

## Additional Information

**Accession codes:** Whole transcriptome sequencing data has been deposited in GEO with accession ID GSE89775. 

**How to cite this article**: Ranganathan, S. *et al*. Loss of EGFR-ASAP1 signaling in metastatic and unresectable hepatoblastoma. *Sci. Rep.* **6**, 38347; doi: 10.1038/srep38347 (2016).

**Publisher's note:** Springer Nature remains neutral with regard to jurisdictional claims in published maps and institutional affiliations.

## Supplementary Material

Supplementary Dataset

Supplelmentary Tables 1–5

## Figures and Tables

**Table 1:** Differences in staining intensity for β-catenin, EGFR, ASAP1 and ERBB4 between fetal, embryonal and SCU tumor cells obtained from 51, 29 and 10 HBL tumors, respectively.

Tumor cell Type	β-catenin				EGFR				ASAP1				ERBB4			
	Unstained		Stained		Unstained		Stained		Unstained		Stained		Unstained		Stained	
	N	%	N	%	N	%	N	%	N	%	N	%	N	%	N	%
Fetal (n = 51)	9	18.0	41	82.0	12	23.5	39	76.5	2	4.0	48	96.0	20	39.2	31	60.8
Embryonal (n = 29)	0	0	29	100	13	44.8	16	55.2	1	3.4	28	96.6	24	82.8	5	17.2
SCU (n = 10)	0	0	10	100	10	100	0	0	6	60.0	4	40.0	10	100	0	0
p-value	0.02*				<0.001				<0.001*				<0.001			

Stained cells show immunostaining intensity of 1+, 2+ and 3+. Unstained cell show staining intensity of zero. P-values are shown for chi-square test. *p-value based on comparison of fetal cells from 50 of 51 tumors.

**Table 2:** Differences in staining intensity for β-catenin, EGFR, ASAP1 and ERBB4 between resectable and unresectable tumors is shown for fetal, embryonal and SCU tumor cells obtained from 51, 29 and 10 HBL tumors, respectively.

Fetal	β-catenin				EGFR				ASAP1				ERBB4			
	Unstained		Stained		Unstained		Stained		Unstained		Stained		Unstained		Stained	
	N	%	N	%	N	%	N	%	N	%	N	%	N	%	N	%
no Tx (n = 34)	4	12.1	29	87.9	7	20.6	27	79.4	1	3.0	32	97.0	11	32.4	23	67.6
Tx/Mets (n = 17)	5	29.4	12	70.6	5	29.4	12	70.6	1	5.9	16	94.1	9	52.9	8	47.1
p-value	0.132* (NS)				0.484 (NS)				0.626* (NS)				0.156 (NS)			
**Embryonal**																
no Tx (n = 17)	0	0	17	100	5	29.4	12	70.6	1	5.9	16	94.1	13	76.5	4	23.5
Tx/Mets (n = 12)	0	0	12	100	8	66.7	4	33.3	0	0	12	100	11	91.7	1	8.3
p-value	NA				0.047				0.392 (NS)				0.286 (NS)			
**SCU**																
no Tx (n = 6)	0	0	6	100	6	100	0	0	2	33.3	4	66.7	6	100	0	0
Tx/Mets (n = 4)	0	0	4	100	4	100	0	0	4	100.0	0	0	4	100	0	0
p-value	NA				NA				0.035				NA			

Stained cells show immunostaining intensity 1+, 2+ and 3+. Unstained cells show staining intensity of zero. P-values are shown for chi-square test. *p-value based on comparison of fetal cells from 50 of 51 tumors.
