# Supplementary material for: Loss of EGFR-ASAP1 signaling in metastatic and unresectable hepatoblastoma
Source: Sci Rep. 2016 Dec 2;6:38347. doi: 10.1038/srep38347 (PMC5133573; doi:10.1038/srep38347)
Supplement: Supplementary Dataset [file srep38347-s1.pdf]

Supplementary information title page:

Loss of EGFR-ASAP1 signaling in metastatic and unresectable hepatoblastoma

Sarangarajan Ranganathan[2*], Mylarappa Ningappa[1*], Chethan Ashokkumar[1], Brandon W. Higgs[1], Jun Min[4], Qing Sun[1], Lori Schmitt[2], Shankar Subramaniam[4], Hakon Hakonarson[3], Rakesh Sindhi[1*]

1. Supplementary Tables 1-5
